# Whittier

**Published:** 1892-11

**Authors:** 


### WHITTIER.

We extract the following exquisite lines from one of Whittier's last poems, which appears in the November *St. Nicholas* Magazine. They are truly prophetic of the end.

"I would not if I could repeat
A life which still is good and sweet;
I keep in age, as in my prime,
A not uncheerful step with time,
And, grateful for all blessings sent,
I go the common way, content
To make no new experiment.
On easy terms with law and fate,
For what must be I calmly wait,
And trust the path I cannot see,—
That God is good sufficeth me.
And when at last upon life's play
The curtain falls, I only pray
That hope may lose itself in truth,
And age in Heaven's immortal youth,
And all our loves and longing prove
The foretaste of diviner love!"

